# Convenience stores are the key food environment influence on nutrients available from household food supplies in Texas Border *Colonias*

**DOI:** 10.1186/1471-2458-13-45

**Published:** 2013-01-17

**Authors:** Joseph R Sharkey, Wesley R Dean, Courtney C Nalty, Jin Xu

**Affiliations:** 1 Program for Research in Nutrition and Health Disparities, Department of Health Promotion and Community Health Sciences, School of Rural Public Health, Texas A&M Health Science Center, College Station, TX, USA

## Abstract

**Background:**

Few studies have focused on the relationship between the retail food environment and household food supplies. This study examines spatial access to retail food stores, food shopping habits, and nutrients available in household food supplies among 50 Mexican-origin families residing in Texas border *colonias*.

**Methods:**

The design was cross-sectional; data were collected in the home March to June 2010 by *promotora*-researchers. Ground-truthed methods enumerated traditional (supercenters, supermarkets, grocery stores), convenience (convenience stores and food marts), and non-traditional (dollar stores, discount stores) retail food stores. Spatial access was computed using the network distance from each participant’s residence to each food store. Data included survey data and two household food inventories (HFI) of the presence and amount of food items in the home. The Spanish language interviewer-administered survey included demographics, transportation access, food purchasing, food and nutrition assistance program participation, and the 18-item Core Food Security Module. Nutrition Data Systems for Research (NDS-R) was used to calculate HFI nutrients. Adult equivalent adjustment constants (AE), based on age and gender calorie needs, were calculated based on the age- and gender composition of each household and used to adjust HFI nutrients for household composition. Data were analyzed using bivariate analysis and linear regression models to determine the association of independent variables with the availability of each AE-adjusted nutrient.

**Results:**

Regression models showed that households in which the child independently purchased food from a convenience store at least once a week had foods and beverages with increased amounts of total energy, total fat, and saturated fat. A greater distance to the nearest convenience store was associated with reduced amounts of total energy, vitamin D, total sugar, added sugar, total fat, and saturated fat. Participation in the National School Lunch Program (NSLP) was associated with lower household levels of total energy, calcium, vitamin C, sodium, vitamin D, and saturated fat. Spatial access and utilization of supermarkets and dollar stores were not associated with nutrient availability.

**Conclusions:**

Although household members frequently purchased food items from supermarkets or dollar stores, it was spatial access to and frequent utilization of convenience food stores that influenced the amount of nutrients present in Texas border *colonia* households. These findings also suggest that households which participate in NSLP have reduced AE-adjusted nutrients available in the home. The next step will target changes within convenience stores to improve in-store marketing of foods and beverages to children and adults.

## Background

Nutrition-related health conditions, such as obesity and type 2 diabetes, are increasing among Mexican-origin children and adolescents [1,2]. Risk factors include increased intakes of energy-dense and nutrient-poor foods, such as fats, salty snacks, desserts, and sugar-sweetened beverages. For poor populations, energy-dense foods may also be more affordable and accessible [3-5]. Social-ecological approaches suggest access to multiple levels of the food environment underpin nutritional health [6]. However, the preponderance of published studies focuses on access to larger stores in the retail food environment, especially supermarkets [3,7-14]. Smaller community and neighborhood retail food venues may exert a greater influence on the acquisition of household food supplies [15,16], especially for limited resource families in rural/underserved areas [3,17]. Neighborhood food stores, such as convenience stores, are ubiquitous, smaller in size and carry fewer fresh and healthier food items [3,18-20].

Household food supplies, which have shown intra-month volatility in presence and amount of specific foods [21], may serve as an intermediary between the retail food environment and dietary intake [15,22,23]. Household availability of energy, nutrients, and foods may be one of the most critical determinants of dietary intakes and a target for intervention work [24-29]. Factors that may influence household food resources include spatial access to traditional, convenience, and non-traditional retail food stores, transportation, timing and amount of household income, cultural repertoires, food-related household technology, and the household environment (e.g., refrigeration and storage) [21,30,31].

Research has examined the neighborhood food environment and its association with dietary intake [32-36], but not its association with household food availability, especially at the nutrient level [30]. Few studies have focused on the relationship between the retail food environment and household food supplies [30]. Thus, our study examined spatial access to traditional, convenience, and non-traditional retail food stores, food shopping habits, and nutrients available in household food supplies among 50 Mexican-origin families that reside in Texas border *colonias*.

## Methods

### Study setting and participants

The study was conducted in *colonias* in Hidalgo County, located in the Lower Rio Grande Valley of Texas along the U.S.-Mexico border. Ground-truthed methods were employed to enumerate the presence of traditional, convenience, and non-traditional retail food stores [3,37]. Trained observers systematically drove all the streets/roads within the study area, identified specific types of traditional (supercenters, supermarkets, and grocery stores), convenience (convenience stores and food marts), and non-traditional retail food stores (dollar stores), and collected data for determination of geographic coordinates.

Study participants were recruited by *promotora*-researchers (indigenous, State of Texas-certified community health workers trained in research methodologies) from spatially-identified areas and provided consent to participate in the study. Details on eligibility (mothers with at least one child 6–11 years living at home) and recruitment have been described elsewhere [38]. All materials and protocols were approved by the Texas A&M University Institutional Review Board.

### Data collection

This analysis focuses on data collected March to June 2010 from all 50 mothers during two in-home visits (two-week interval): survey data from the first visit and two household food inventories (HFI). All survey data were collected from the mother in Spanish, the language spoken in all participants’ homes. The interviewer-administered survey included demographics (age, education, self-identified ethnicity, marital status, nativity, household composition, and household income), access to transportation (car ownership and car availability during the day), food purchasing from a main grocery store (frequency), dollar or discount store (frequency), and convenience store (the frequency at which food was purchased for the household, the frequency food was purchased food for child, and the frequency at which the child purchased food on his/her own), food and nutrition assistance program participation (Supplemental Nutrition Assistance Program [SNAP], Special Supplemental Nutrition Program for Women, Infants and Children [WIC], School Breakfast Program [SBP], and National School Lunch Program [NSLP]), and the 18-item Core Food Security Module, which classified households as being food secure, moderately food secure, low food secure, or very low food secure [39].

#### Household food inventory (HFI)

The HFI, which included 252 items, was modified for a prior HFI project in Texas border *colonias* to include regional and cultural food items [21]. *Promotora*-researchers received two full days of training and pretesting prior to completing two detailed inventories of household food supplies (two-week interval) in the homes of study participants. The HFI documented the presence and amount (number or weight) of the following food categories: *verdura fresca* (fresh vegetables), *fruta fresca* (fresh fruit), *cereales* (cereals), *pan, pasteles, galletas saladas y galletas* (bread, cakes, crackers, and cookies), *tortillas, pasta y arroz* (tortillas, pasta and rice), *leche*, *lechería, helado, yogur y queso* (milk, dairy, ice cream, yogurt, and cheese), *carne, carne de aves, jamóny salchichas, frescas o congeladas* (fresh or frozen meat, poultry, ham and sausage), *mariscos, frescos, congelados o enlatados* (fresh, frozen or canned seafood,*palomitas de maiz o papitas* (popcorn and chips), *legumbres* (legumes), *verduras enlatadas/frasco* (canned or jarred vegetables), *frutas enlatadas* (canned fruit), *sopas y consomes* (soups and broths), *bebidas* (beverages), *artículos de comida miscelánea* (miscellaneous pantry items that excluded commercial baby food, infant formula, and condiments), *verduras congeladas* (frozen vegetables), *frutas congeladas* (frozen fruit), *aceites y otras mantecas* (oils and other fats). Canned fruit was identified as being packed in *sirope espeso* (heavy syrup) or *sirope/jugo ligero* (light syrup or juice). Hard copy HFI data were entered into Nutrition Data Systems for Research (NDS-R) 2009 for nutrient analysis. Two-time mean nutrient availability, with equal weighting for each of the two HFI, were calculated for each household for total energy, protein, dietary fiber, calcium, potassium, sodium, vitamin C, vitamin D, total sugars, added sugars, total fat, and saturated fat.

*Spatial access* was computed using a measure of network distance along the road network; network distance was calculated with ESRI’s Network Analysis extension in ArcInfo 9.2 from each participant’s residence to the geographic position in front of each food store. Two criteria of access were calculated for each participant: 1) proximity (minimum network distance) to nearest retail food stores by type, and 2) coverage (number of different retail food stores by type within one and three network mile buffers) [40]. The buffer distances represent the areas of a long walk (one mile) and reachable by car (within 3 miles), and were used in prior work in Texas border *colonias*[3].

### Statistical analysis

All statistical analyses were conducted with Release 12 of Stata Statistical Software (College Station, TX). Descriptive statistics were calculated for demographics, access to transportation, food purchasing, food and nutrition assistance program participation, the 18-item Core Food Security Module, and spatial access to food stores (proximity and coverage). Nutrient availability in household food supplies was calculated using an adult-equivalent adjustment for household composition. To account for gender- and age-specific differences in calorie needs, adult-equivalent adjustment constants were calculated using MyPyramid Food Intake Pattern Calorie Levels [41] as follows: 1) calorie level for reference group was calculated by averaging the MyPyramid calorie levels for males 19-50 years, using sedentary and moderate activity levels; 2) gender-specific levels were calculated for specific age groups (2–3 y, 4–8 y, 9–13 y, 14–18 y, 19–50 y, 51–60 y, and >60 y); and 3) adult-equivalent constants were calculated as a ratio between gender- and age-specific calorie levels and the 19–50 y male reference. Total adult equivalents were calculated for each household by summing the adult-equivalent adjustments for the household members (children and adults). The adult-equivalent (AE)-adjusted household food availability was calculated by dividing the average of the two HFIs by the household total adult equivalents. Bivariate correlations with AE-adjusted household nutrient availability were estimated using Pearson’s product–moment correlation. Separate linear regression models were estimated to determine the association of independent variables with the availability of each AE-adjusted nutrient. Statistically significant (p < 0.05) variables from the bivariate correlation estimations were simultaneously entered. Backward elimination strategy was used to sequentially remove statistically non-significant variables to obtain the “best” set of independent variables [42].

## Results

Table 1 shows descriptive statistics for the sample, based on the mothers’ responses to the Spanish-language survey. All participants considered themselves to be Hispanic or Mexican; 94% were born in Mexico. Thirty-nine women reported their household income below 50% of the Federal Poverty Level (FPL), with another 16% at 50-74% FPL. Less than 60% of the households participated in the National School Lunch Program (NSLP). Almost three-fourths of the households were considered low or very low food secure − formerly known as food insecure with hunger. Sixty percent of the mothers used a main grocery store at least once a week and 30% purchased food from a dollar or discount store at least once a week. Convenience stores were a frequent source of food items (at least once a week). Forty-four percent did not have a car available during the day, 84% reported purchasing food from a convenience store for their child and 26% reported their child purchased food from a convenience store on their own at least once a week.

**Table 1:** **Characteristics of participant households (*n *= 50), using mothers’ responses to in-home survey**

	**Mean ± SD**	**% (*n*)**
Age, y^a^	35.2 ± 7.0	
Education, y	8.5 ± 3.1	
Ethnicity^b^		
Hispanic		32 [16]
Mexican		68 [34]
Married^c^		82 [41]
Country of birth		
Mexico		94 (47)
Household composition^d^	5.7 ± 1.5	
Household income, % FPL^e^		
<50		78 [39]
50–74		16 [8]
75–99		2 [1]
100–130		4 [2]
Car ownership		80 [40]
Car availability during the day		56 [28]
Nutrition program participation		
SNAP		88 (44)
WIC		58 [29]
SBP		96 (48)
NSLP		58 [29]
Food security status^f^		
Food secure		6 [3]
Moderate food security		20 [10]
Low food security		40 [20]
Very low food security		34 [17]
Use of main grocery store		
At least once/week		60 [30]
Use of dollar store for food		
At least once/week		30 [15]
Use of convenience store		
Eat food from convenience store at least once/week		78 [39]
Purchase food from convenience store for child at least once/week		84 [42]
Child purchases food from convenience store on their own at least once/week		26 [13]

NSLP = National School Lunch Program; SNAP = Supplemental Nutrition Assistance Program; SBP = School Breakfast Program; WIC = Special Supplemental Program for Women, Infants and Children.

^a^ as of June 2010.

^b^ self-identified.

^c^ Married or living with a partner.

^d^ Total of adults and children living in the household.

^e^ Based on 2010 criteria using household income and household composition.

^f^ Based on 18-item USDA Food Security Module.

The average of two household food inventories (HFI) was used to calculate the overall availability of nutrients from household food supplies. Nutrient availability within each household was adjusted using adult-equivalent constants for household members. The mean total adult equivalents in this sample was 4.11 (SD = 1.04), median = 3.97, range 2.11-6.93. Unadjusted and AE-adjusted nutrients available in the homes are shown in Table 2. Using measures of proximity (distance to nearest store) and coverage (number of different stores within one or three miles from each residence), there was significantly greater access to convenience stores (*p* <0.001) than any other type of food store (Table 3). In bivariate correlations (data not shown), distance to the nearest convenience store was the only proximity measure associated with the AE-adjusted household food supply (total energy, sodium, vitamin D, total sugars, added sugar, total fat, and saturated fat); proximity to the nearest traditional or non-traditional food store was not correlated with any of the household nutrients. Similarly, there was no correlation between coverage of traditional or non-traditional food stores and household nutrients. However, coverage measures for convenience stores were correlated with household availability of vitamin D, total sugars, and added sugar. Participation in WIC was positively associated with Vitamin D, while participation in NSLP was associated with lower AE-adjusted household levels of total energy, protein, dietary fiber, calcium, potassium, vitamin C, sodium, vitamin D, total fat, and saturated fat. None of the food security categories were associated with household availability of any of the nutrients. Among shopping behaviors, only child’s purchase of food from a convenience store on their own was positively associated with household availability of energy, protein, fat, and saturated fat.

**Table 2:** **Nutrient availability in 50 households based on two household food inventories – unadjusted and AE-adjusted**^**1**^

	***Household nutrient availability***		***AE-Adjusted household nutrient availability***	
	Mean ± SD	Median	Mean ± SD	Median
Energy, kcal	119,899 ± 37,678	117,320	30,566.3 ± 10,992.9	30,002.9
Protein, g	3,363.4 ± 1,180.7	3,181.7	865.0 ± 373.9	832.4
Dietary fiber, g	681.2 ± 354.9	943.7	256.0 ± 126.3	235.2
Calcium, mg	20,516.9 ± 7,057.4	20,531.7	5,307.4 ± 2,271.7	4,704.1
Potassium, mg	108,649.7 ± 42,659.4	99,325.3	28,225.4 ± 13,399.9	25,688.4
Sodium, mg	110,220.5 ± 40,885.3	106,262.4	28,600.4 ± 12,965.3	28,416.6
Vitamin C, mg	2,964.4 ± 1,370.4	2,570.3	767.4 ± 400.0	673.5
Vitamin D, μg	184.2 ± 64.4	181.1	47.7 ± 20.7	44.8
Total sugars, g	2,920.9 ± 1,288.2	2,766.5	750.5 ± 341.8	712.3
Added sugars, g	2,133.8 ± 1,240.3	1,692.4	541.2 ± 294.6	501.8
Total fat, g	6,911 ± 2,559.2	6,905.4	1,753.7 ± 691.6	1,722.3
Saturated fat, g	2,026.1 ± 748.1	2,018.4	515.6 ± 206.0	504.3

^1^ AE-Adjusted (Adult-Equivalent Adjusted) using adult-equivalent adjustment constants for each household.

**Table 3:** **Spatial accessibility to food for the participant households (*n *= 50)**

	**Mean ± Standard Deviation**	**Median**
***Proximity (distance in miles)***^***a***^		
Traditional Food Stores		
Supermarkets	5.0 ± 2.5	5.5
Convenience		
Convenience stores/food marts	0.75 ± 0.56	0.67
Non-Traditional Food Stores		
Dollar Stores	3.1 ± 1.8	2.3
***Coverage – 1 Mile***^***b***^		
Traditional Food Stores		
Supermarkets	0.06 ± 0.24	0
Convenience		
Convenience stores/food marts	3.3 ± 2.4	2.5
Non-Traditional Food Stores		
Dollar Stores	0.1 ± 0.3	0
***Coverage – 3 Miles***^***c***^		
Traditional Food Stores		
Supermarkets	1.0 ± 1.3	0
Convenience		
Convenience stores/food marts	25.6 ± 8.6	29.5
Non-Traditional Food Stores		
Dollar Stores	1.7 ± 1.6	1.5

^a^ Network distance calculated from the residence of each of the 50 participant families to the nearest food store.

^b^ Number of different food stores by store type within 1 network mile of the residence.

^c^ Number of different food stores by store type within 3 network miles of the residence.

Estimates from AE-adjusted linear regression models are shown in Table 4. Participation in the NSLP was associated with lower AE-adjusted levels of total energy, calcium, vitamin C, sodium, vitamin D, and saturated fat. Child’s purchasing of foods from convenience stores at least once a week was associated with greater household levels of total energy, total fat, and saturated fat. Distance to the nearest convenience store was associated with household availability of nutrients. Greater distance was significantly associated with lower availability of total energy, vitamin D, total sugar, added sugar, total fat, and saturated fat.

**Table 4:** Association of sample characteristics and spatial access to convenience stores with adult-equivalent adjusted household availability of nutrients from food

	**Energy (kcal)**	**Protein (g)**	**Dietary Fiber (g)**	**Calcium (mg)**	**Potassium (mg)**	**Vitamin C (mg)**	**Sodium (mg)**	**Vitamin D (μg)**	**Total Sugar (g)**	**Added Sugar (g)**	**Total Fat (g)**	**Total SFA (g)**
NSLP^a^	−6228.2^*^	−213.5	−63.4	−1366.7^*^	−5570.1	−328.7^**^	−9506^**^	−14.9^**^	−69.0	−10.7	−334.6	−130.2^*^
Convenience – Child^b^	8081.8^**^	234.7	35.2	694.9	6364.3	4.3	5361.5	3.8	97.4	45.5	620.1^***^	138.8^*^
Convenience Store^c^	−6767.3^**^	−139.8	−35.7	−520.1	−4751.2	−50.7	−5214.8	−9.9^**^	−266.7^***^	−233.8^***^	−381.5^**^	−124.4^***^
*R*^*2*^	0.343	0.234	0.122	0.147	0.145	0.184	0.259	0.248	0.228	0.202	0.340	0.348

SFA = Saturated Fatty Acid.

^a^ Participate in the National School Lunch Program.

^b^ Child purchases food from a convenience on own at least once a week.

^c^ Network distance from the residence to the nearest convenience store.

Statistically significant at ^*^p < 0.05 ^**^p < 0.01 ^***^p < 0.001.

## Discussion

This study builds on prior work which demonstrated that convenience stores provide greater spatial access (distance and number of shopping opportunities) than traditional food stores [3,12], and convenience stores expose children and families to a larger assortment of less-healthy foods and beverages compared to healthier options [19]. However, few studies have attempted to examine the relationship between retail food stores and the availability of nutrients in the household. Findings from the current study expand the understanding of the relationship between the local food environment and household nutrient availability. This is apparently the first study to document the relationships of food store access, food shopping behaviors, food and nutrition assistance programs, and AE-adjusted nutrients present in the household.

There are a number of key findings. First, spatial access, in terms of proximity (distance to the nearest food store), was significant only for convenience stores, not for traditional (supercenters, supermarkets, or grocery stores) or non-traditional food stores (dollar or discount stores). A greater distance to the nearest convenience store was associated with reduced household amounts of total energy, vitamin D, total sugar, added sugar, total fat, and saturated fat. Second, frequency of shopping at a main grocery store or dollar/discount store was not associated with household food supplies; however, households in which the child purchased food from a convenience store on his/her own at least once a week had increased amounts of total energy, protein, sodium, fat, and saturated fat. Finally, NSLP participation was associated with lower amounts of present nutrients compared to households that did not participate in the NSLP. This suggests that households that participate in NSLP may reduce their household food supplies since meals are provided for their children in school. It could also be that despite NSLP participation, households are still nutrient poor, in the sense that it is helpful to participating children, but it does not extend to other household members. Overall, these findings document that access and utilization of convenience stores reflect nutrient availability in household food supplies among limited-resource families living in Texas border *colonias*.

The findings are relevant, given that these areas have limited access to supermarkets or stores that offer a variety of foods and residents thus rely on small stores that are conveniently located for frequent replenishment of food and beverage items [3,21,43]. It is important to keep in mind that convenience stores are a valuable element of the food environment that provide children and families with increased exposure to sugar-sweetened beverages and snack foods [19]. Interestingly, food security and household income were not associated with household food supplies, and were dropped from the final models. The one non-convenience store food source that was associated with household nutrient availability was the National School Lunch Program and its important role in bolstering household food supplies among vulnerable populations should be emphasized and further examined in future research.

There are a number of strengths to this study, including understanding the experiences of a hard-to-reach Mexican-origin sample, use of Spanish-language data collected in the home by trained *promotora*-researchers, nutrient analyses from multiple, comprehensive inventories of household food supplies, AE-adjustment of household supplies, and ground-truthed identification and geocoding of traditional, convenience, and non-traditional retail food stores. To our knowledge, this is the first study to examine a relationship between access to and utilization of convenience stores and nutrient availability within home food supplies. However, there are a number of limitations that warrant mention, such as small sample size, cross-sectional study design, and lack of seasonal variation. Despite such limitations, the research is a valuable contribution to the literature and researchers’ and policy-makers’ understanding of the role of convenience stores on household food supplies among Mexican-origin *colonia* dwellers.

## Conclusion

The results of this study further our understanding of the retail food environment’s influence on household food supplies, an important mediator between the retail food environment and dietary intake. Results indicate convenience stores are an important food source among limited resource Mexican-origin families who are at great risk for poor nutrition and nutrition-related health conditions. Now considered to be a major part of the food environment, convenience stores or food marts offer increased access to beverages and food items, especially in rural and underserved areas [19]. These food venues provide culturally-accepted food with greater convenience than traditional and non-traditional food stores. The next step will be to determine what physical, economic, and sociocultural changes within small stores need to be made in order to improve the food environment at large as well as the nutritional content of household food supplies for *colonia* residents.

## Abbreviations

FPL: Federal Poverty Level; NSLP: National School Lunch Program; SFA: Saturated Fatty Acid; SNAP: Supplemental Nutrition Assistance Program; WIC: Special Supplemental Nutrition Program for Women, Infants and Children.

## Competing interests

The authors declare that they have no competing interests.

## Authors’ contributions

JRS designed the study, and worked on the development of the instrument and the protocol for collection of data. JRS and WRD wrote the first draft of the paper. JX calculated the AE-adjustment constants. JRS, WRD, CCN, and JX read and approved the final manuscript.

## Pre-publication history

The pre-publication history for this paper can be accessed here:

http://www.biomedcentral.com/1471-2458/13/45/prepub
